# Colorectal cancer mortality and industrial pollution in Spain

**DOI:** 10.1186/1471-2458-12-589

**Published:** 2012-08-01

**Authors:** Gonzalo López-Abente, Javier García-Pérez, Pablo Fernández-Navarro, Elena Boldo, Rebeca Ramis

**Affiliations:** 1 Cancer and Environmental Epidemiology Unit, National Centre for Epidemiology, Carlos III Institute of Health, Monforte de Lemos 5, 28029, Madrid, Spain; 2 Consortium for Biomedical Research in Epidemiology & Public Health (CIBER en Epidemiología y Salud Pública - CIBERESP), Madrid, Spain

**Keywords:** Epidemiology, Colorectal neoplasms, Industrial pollution, Environmental pollution/prevention and control

## Abstract

**Background:**

Records kept as a result of the implementation of Integrated Pollution Prevention and Control (IPPC) and the European Pollutant Release and Transfer Register (E-PRTR) constitute a public inventory of industries, created by the European Commission, which is a valuable resource for monitoring industrial pollution. Our objective is to ascertain whether there might be excess colorectal cancer mortality among populations residing in the vicinity of Spanish industrial installations that are governed by the IPPC Directive and E-PRTR Regulation and report their emissions to air.

**Methods:**

An ecological study was designed to examine colorectal cancer mortality at a municipal level (8098 Spanish towns), over the period 1997–2006. We conducted an exploratory "near vs. far" analysis to estimate the relative risks (RR) of towns situated at a distance of less than 2 km from industrial installations. The analysis was repeated for each of the 24 industrial groups. RR and their 95% credible/confidence intervals (95%CI) were estimated on the basis of Poisson regression models, using two types of modelling: a) the conditional autoregressive Bayesian model proposed by Besag, York and Mollié, with explanatory variables; and b) a mixed regression model. Integrated nested Laplace approximations were used as a Bayesian inference tool.

**Results:**

Statistically significant RRs were detected in the vicinity of mining industry (RR 1.258; 95%CI 1.082 - 1.463), paper and wood production (RR 1.071; 95%CI 1.007 – 1.140), food and beverage sector (RR 1.069; 95%CI 1.029 - 1.111), metal production and processing installations (RR 1.065; 95% CI 1.011 – 1.123) and ceramics (RR 1.050 ; 95%CI 1.004 – 1.099).

**Conclusions:**

Given the exploratory nature of this study, it would seem advisable to check in other countries or with other designs, if the proximity of industries that emit pollutants into the air could be an added risk factor for colorectal cancer mortality. Nevertheless, some of the differences between men and women observed in the analyses of the industrial groups suggest that there may be a component of occupational exposure, little-studied in the case of cancers of the digestive system.

## Background

Colorectal cancer (CRC) is the fourth leading cancer affecting both sexes worldwide. Incidence and mortality vary widely among countries, depending on their degree of development. The highest incidence rates are registered in Australia/New Zealand, North America and Europe, and are practically double those of Asia and South America. In 2008, estimates put the global number of incident cases at 1,233,000, with developed countries accounting for 726,000 (60%) of this total [1]. In Spain, 1 out of every 7 cancer-related deaths in 2008 was due to colorectal neoplasm, thus making it the second leading cancer among men (after lung cancer) and women (after breast cancer), accounting for a total of 13,793 deaths.

In Spain [2] and other developed countries [3] there has been an increase in incidence due to this type of cancer, which has been attributed to changes in the dietary habits of the Spanish population, including higher consumption of sugar and red and processed meat, lower consumption of fibre [4], and less physical activity. This incidence trend is in sharp contrast to that of mortality, inasmuch as the latter changed in 1997–1998 and led to a subsequent decline in mortality rates across the sexes [2].

Non-dietary causes of CRC for which there is evidence include genetic predisposition (hereditary non-polyposis colorectal cancer (HNPCC), hereditary polyposis, and polymorphisms in other enzymatic systems), and ulcerative colitis, in which aspirin and other anti-inflammatories serve to reduce the risk [5]. Little attention has been paid to factors, such as occupational exposures and environmental and industrial pollution, exposures which have evolved in parallel to the incidence of these tumours, with publications on the topic being few and, in many cases, rather inconclusive.

Occupational agents showing some evidence of an association with CRC incidence include asbestos [6], though this association has been questioned [7,8], and some types of metalworking fluids (a range of oils and other chemical substances used to cool and/or lubricate metal work-pieces) which have been associated with rectal cancer in exposed workers [9,10]. In addition, many of the pollutants released by industries are carcinogens, and some of these have been associated with tumours of the digestive system [11]. Occupations too have been described as being specifically associated with tumours of the colon and rectum incidence [12]. Insofar as general population exposure to industrial pollution is concerned, an earlier exploratory study has reported that mortality due to tumours of the digestive system might be higher among populations residing in the vicinity of industrial sources in the metal sector than in more distant or unexposed populations [13,14], all of which goes to underscore the interest in studying the possible relationship between industrial pollution and CRC.

European Commission directives passed in 2002 afforded a new means of studying the consequences of industrial pollution: Integrated Pollution Prevention and Control (IPPC), governed both by Directive 96/61/CE and by Act 16/2002 which incorporates this Directive into the Spanish legal system, lays down that, to be able to operate, industries covered by the regulation must obtain the so-called Integrated Environmental Permit. The application of this measure has entailed the creation of the new European Pollutant Release and Transfer Register (E-PRTR) to which all emissions of a wide list of pollutants must be compulsorily reported. IPPC and E-PRTR records thus constitute an inventory of industries, which facilitates the monitoring of industrial pollution and, by extension, renders it possible for the association between residential proximity to such pollutant installations and risk of cancer mortality to be studied [15-17] .

The aim of this study was to ascertain whether the population that resides in the vicinity of Spanish industrial installations governed by the IPPC Directive and E-PRTR Regulation might display excess CRC mortality.

## Methods

A mortality study was designed, with death data being sourced from the registers of the National Statistics Institute (*Instituto Nacional de Estadística* - *INE*) and exposure data being drawn from the E-PRTR. The cause of death studied were those coded as malignant neoplasm of colon, rectum and anus, codes 153–154, 159.0 (International Classification of Diseases-9^th^ Revision/ICD-9) and C18–C21, C26.0 (ICD-10). The designated study period was 1997–2006, and Spanish towns were taken as the spatial unit of analysis. By way of reference for calculating expected CRC cases in each town, we used the overall rates for Spain, broken down by age group, sex and period (18 age groups: 0–4, 5–9,…,85 and over; and two five-year periods 1997–2001, 2002–2006), and the person-years for each town in the two periods considered.

With respect to exposure, data on industrial pollutant sources for 2007 were obtained from the E-PRTR and IPPC registries and supplied to us by the Spanish Ministry for the Environment and Rural & Marine Habitats. Population exposure to industrial pollution was estimated by reference to the distance from town centroids to industrial facilities. This meant that all the geographic co-ordinates of the industries registered had to be validated using orthophotos and detailed information obtained with the aid of the new tools provided by Internet and Google Earth (with aerial images and street view application). Some of these validation procedures have been described elsewhere [18].

To estimate the relative mortality risk in towns lying at different distances from any given installation, we conducted a “near vs. far” analysis of the respective town's proximity to the source of risk. The choice of the distance shown in the results (near: 2 km or less from any installation) was based on a sensitivity analysis for 2, 3, 4 and 5 kilometres (see Additional file 1). The analysis was repeated for each industrial group. These groups were formed on the basis of the similarity of their air-pollutant emission patterns, and their PRTR-defined codes (Official Government Gazette 2007-*BOE*) are shown in Table 1.

**Table 1:** Industrial groups used. Number of installations, towns lying within <2 km and amounts of selected carcinogenic pollutants released to air (in Tn) (Spain 2007)

	**E-PRTR category**	**No. of ind. facilities**	**Towns <2 km**	**Arsenic**	**Benzene**	**Cadmium**	**Cobalt**	**Chrome**	**Nickel**	Dioxins (kg)*	**Lead**	**Tetra-chloro ethylene**
1 Combustion installations	1.c	138	43	2.639	13.107	0.718	0.026	3.963	18.052	0.001	3.616	0.807
2 Refineries and coke ovens	1.a, 1.d	12	5	0.313	24.193	0.457	85.70	1.858	41.130		0.927	
3 Production and processing of metals	2.a, 2.b, 2.c.i, 2.c.ii, 2.d, 2.e	172	98	3.630	62.949	1.263		5.903	1.730	0.485	37.671	
4 Galvanisation	2.c.iii	36	22		0.025	0.011		0.003	0.040		0.523	
5 Surface treatment of metals and plastic	2.f	248	156	0.008	0.635	0.031		1.749	4.581		0.235	11.711
6 Mining industry	3.a, 3.b	33	14									
7 Cement and lime	3.c, 3.d	70	35	0.345	38.359	0.261	0.044	0.791	0.838	0.001	1.821	
8 Glass and mineral fibres	3.e, 3.f	56	20	0.536	1.647	0.178	0.006	3.546	2.471	0.300	8.139	
9 Ceramics	3.g	457	159	0.479	16.797	0.922	0.015	1.598	6.008		10.661	
10 Organic chemical industry	4.a	149	77	0.051	68.357	0.016	0.026	1.294	0.566	0.001	0.245	3.802
11 Inorganic chemical industry	4.b	70	28	0.001	0.059	0.023		0.011	0.142		0.244	
12 Fertilisers	4.c	23	19	0.003	0.009	0.003	0.010	0.006	0.110		0.004	
13 Biocides	4.d	12	8									
14 Pharmaceutical products	4.e	55	35				0.008	0.001				0.185
15 Explosives and pyrotechnics	4.f	58	28									
16 Hazardous waste	5.a, 5.b	90	42	0.055		0.032	0.012	0.120	0.145	0.010	0.309	0.062
17 Non-hazardous waste	5.c, 5.d	144	29	0.010	0.151	0.004		0.008	0.020	0.205	0.004	0.932
18 Disposal or recycling of animal waste	5.e	38	22	0.006	0.001	0.004		0.021	0.196		0.009	
19 Urban waste-water treatment plants	5.f, 5.g	86	32		0.177							
20 Paper and wood production	6.a, 6.b, 6.c	88	70	0.028	2.407	0.048		0.232	1.613		0.105	
21 Pre-treatment or dyeing of textiles	9.a	25	24		0.081							
22 Tanning of hides and skins	9.b	2	2									
23 Food and beverage sector	8.a, 8.b, 8.c	310	157	0.015	0.009	0.017	0.019	0.011	0.814		0.018	
24 Intensive rearing of poultry or pigs	7.a	1783	-		0.002							
26 Surface treatment using organic solvents	9.c	80	48	0.001	0.008	0.010		0.053	0.048		0.007	0.009
27 Production of carbon or electro-graphite	9.d	2	0									
28 Ship building	9.e	8	4					0.006				

The exposure variable for each industrial group was coded in the following three levels: 1) reference level, i.e., unexposed towns, defined as population centres having no registered pollutant industries less than 2 km from their municipal centroid; 2) an intermediate group, defined as towns having some type of pollutant industry nearby which did not belong to the group being studied; and 3) an exposed group, i.e., towns having their municipal centroid at a distance ≤2 km from an installation belonging to the group in question. This form of coding meant that the “unexposed” group could be a “clean” group with respect to industrial pollution in general.

RRs and their 95% credible/confidence intervals (95% CIs) for the exposed versus the unexposed groups were estimated on the basis of Poisson regression models, using two types of modelling: a) a Bayesian conditional autoregressive model proposed by Besag, York and Mollié (BYM) [19], with explanatory variables; and b) a mixed regression model. In both cases, observed deaths were the dependent variable and expected deaths were the offset. All estimates for the variable of exposure, described above, were adjusted for the following standardised socio-demographic indicators, chosen for their availability at a municipal level and potential explanatory ability vis-à-vis certain geographic mortality patterns: population size; percentage of illiteracy, farmers and unemployed; average persons per household according to the 1991 census; and, mean income as a measure of income level [20]. The variable of exposure and potential confounding covariates were fixed-effects terms in the models.

In the BYM Bayesian autoregressive model, the random effects terms include two components: a spatial term containing municipal contiguities; and the municipal heterogeneity term. Integrated nested Laplace approximations (INLAs) were used as a tool for Bayesian inference. For the purpose, we used R-INLA [21] with the option of Gaussian estimation of the parameters, a package available in the R environment [22]. A total of 8,098 towns were included, and the spatial data on municipal contiguities was obtained by processing the official INE maps.

The Poisson regression mixed model [23,24] includes province as a random effects term, to enable geographic variability to be taken into account and unexposed towns belonging to the same geographic setting to be considered as the reference level, something that is justified by the geographic differences observed in mortality attributable to these tumours [25].

The results of estimates from the two types of models are shown graphically as forest-plots for ease of interpretation. Of the industrial groups shown in Table 1, we analysed a total of 24, excluding groups 22, 25 and 27 owing to the low number of sources, and group 24 due the fact that it corresponded to farms of little interest for the purposes of our study. A category defined as “Industry” was also included in the figures, representing municipal proximity to some type of pollutant industry, regardless of the industrial group. No account was taken of induction periods because the year of commencement of industrial operations was unknown to us at the date of analysis.

## Results

Table 1 shows the groups of industrial sectors used, along with the number of pollutant sources and respective amounts of IARC group-1 carcinogens released, expressed in tonnes (except for dioxins which are expressed in kilogrammes). Table 2 lists the amounts of pollutants shown by the IPPC as having been released to air, with a note of those belonging to any IARC carcinogen group. There is no scientific evidence to show that listed carcinogens are associated with colorectal neoplasms in humans, perhaps with the exception of dioxins, furans and polychlorinated biphenyls, regarded as multi-site carcinogens and a source of food contamination [26].

**Table 2:** Pollutants released by PRTR-registered industries, amounts in tonnes (Tn) and number of industrial sources reporting these releases (Spain 2007)

**Pollutant**	**CAS number**^**a**^	**IARC Group**^**b**^	**Tn.**	**No. of ind. facilities**
1,1,1-trichloroethane	71-55-6	3	0.99	62
1,2,3,4,5,6- hexachlorocyclohexane(HCH)	608-73-1	2B	0.05	4
1,2-dichloroethane (ethylene dichloride-EDC)	107-06-2	2B	2.63	46
Ammonia (NH3)	7664-41-7		34099.48	2204
Antimony and compounds (expressed as Sb)			75.07	128
Anthracene	120-12-7	3	0.25	32
Arsenic and compounds (expressed as As)	7440-38-2	1	8.12	763
Benzene	71-43-2	1	228.97	581
Cadmium and compounds (expressed as Cd)	7440-43-9	1	4.00	814
Total organic carbon (TOC) (expressed as total C)			8376.12	83
Hydrogen cyanide (HCN)	74-90-8		23.21	64
Chlorofluorocarbons (CFCs)			0.22	2
Chlorine and inorganic compounds (expressed as HCl)			4132.04	545
Vinyl chloride	75-01-4	1	62.83	7
Cobalt and compounds (expressed as Co)	7440-48-4	2A	85.87	134
Copper and compounds (expressed as Cu)	7440-50-8		31.63	756
Non-methane volatile organic compounds (NMVOC)			91368.47	1412
Chromium and compounds (expressed as Cr)	1^c^	21.17	866	
Dichloromethane (DCM)	75-09-2	2B	481.33	36
Carbon dioxide (CO2)	124-38-9		180383930.96	2048
Fluorine and inorganic compounds (expressed as HF)			3960.55	424
Di(2-ethylhexyl)phthalate (DEHP)	117-81-7	3	0.37	10
Hexachlorobenzene (HCB)	118-74-1	2B	0.44	12
Total polycyclic aromatic hydrocarbons PRTR (PRTR total PAHs)			20.64	508
Hydrochlorofluorocarbons (HCFCs)			3.97	16
Hydrofluorocarbons (HFCs)			107.98	34
Manganesium and compounds (expressed as Mn)	7439-96-5		46.42	140
Mercury and compounds (expressed as Hg)	7439-97-6	3	4.91	855
Methane (CH4)	74-82-8		227734.73	2432
Carbon monoxide (CO)	630-08-0		443024.58	1943
Naphthalene	91-20-3	2B	3.95	44
Nickel and compounds (expressed as Ni)	7440-02-0	1	78.51	857
Ethylene oxide	75-21-8	1	14.96	4
Nitrous oxide (N2O)	10024-97-2		6958.52	2214
Sulphur oxides (SOx/SO2)		3	988631.45	1623
Nitrogen oxides (NOx/NO2)			547652.67	2169
Particulate matter (PM10)			39812.78	1427
Total suspended particles (TSP)			18151.73	357
PCDD + PCDF (dioxins + furans) (expressed as Teq)		1	0.001	441
Pentachlorophenol (PCP)	87-86-5	2B	0.06	3
Perfluorocarbons (PFCs)			21.91	5
Lead and compounds (expressed as Pb)	7439-92-1	2A	64.54	805
Polychlorinated biphenyls (PCBs)	1336-36-3	2B	0.03	18
Talium and compounds (expressed as Tl)	7440-28-0		0.26	18
Tetrachloroethylene (PER)	127-18-4	2A	17.51	17
Tetrachloromethane (TCM)	56-23-5		0.94	44
Trichlorobenzenes (TCBs) (all isomers)	12002-48-1		0.42	4
Trichloroethylene	79-01-6	2A	58.64	42
Trichloromethane	67-66-3		13.17	18
Vanadium and compounds (expressed as V)	7440-62-2		453.64	70
Zinc and compounds (expressed as Zn)	7440-66-6		261.05	764

^a^ When the pollutant is a group of substances, the CAS is not specified.

^b^ IARC carcinogen classification: Group 1 (carcinogenic to humans); Group 2A (probably carcinogenic to humans); Group 2B (possibly carcinogenic to humans); Group 3 (not classifiable in terms of carcinogenicity to humans).

^c^ Hexavalent chromium is Group 1, the remainder are Group 3.

**Figure 1:**
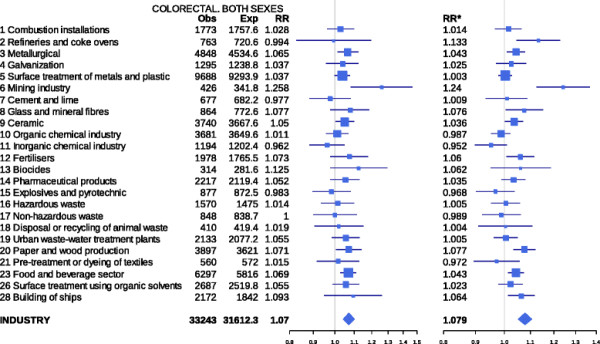
**Colorectal cancer mortality in towns situated near pollutant industries belonging to different industrial groups. Both sexes. Spain 1997–2006.** Observed (Obs) and expected (Exp) deaths in towns lying 2 km or less from pollutant industries, relative risks obtained with the two models used, and 95% credible (BYM model) and confidence intervals (mixed model). RR = Relative risk BYM model. RR* = Relative risk Poisson regression mixed model.

**Figure 2:**
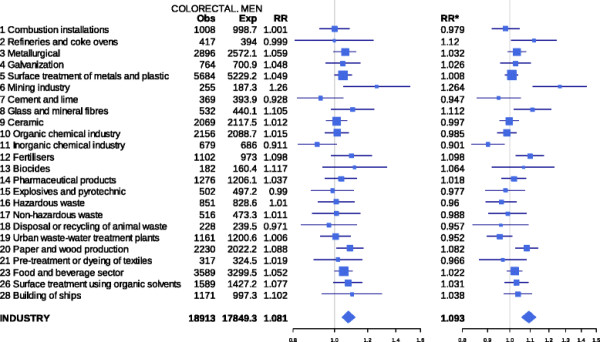
**Colorectal cancer mortality in towns situated near pollutant industries belonging to different industrial groups. Men. Spain 1997–2006.** Observed (Obs) and expected (Exp) deaths in towns lying 2 km or less from pollutant industries, relative risks obtained with the two models used, and 95% credible (BYM model) and confidence intervals (mixed model). RR = Relative risk BYM model. RR* = Relative risk Poisson regression mixed model.

**Figure 3:**
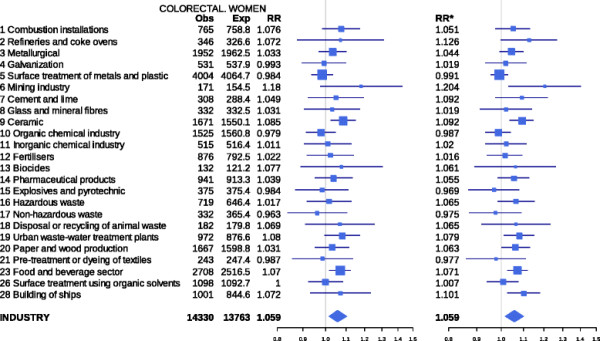
**Colorectal cancer mortality in towns situated near pollutant industries belonging to different industrial groups. Women. Spain 1997–2006.** Observed (Obs) and expected (Exp) deaths in towns lying 2 km or less from pollutant industries, relative risks obtained with the two models used, and 95% credible (BYM model) and confidence intervals (mixed model). RR = Relative risk BYM model. RR* = Relative risk Poisson regression mixed model.

There were 120,841 deaths due to CRC in the study period. Figure 1 depicts the results for both sexes of the analyses performed by type of industry, using the two regression models. For each industrial group the figure shows: observed and expected cases in towns situated at 2 km or less from pollutant industries; the RRs obtained with the two estimates; and the 95% credible intervals (BYM model) and confidence intervals (mixed model). Figures2 and 3 depict the results for men and women respectively. The results indicate that, across the sexes, populations residing ≤ 2 km from pollutant facilities faced a higher risk than did unexposed or distant populations (7.1% for the BYM model and 7.9% for the mixed model).

The highest statistically significant RRs in the joint analysis of both sexes (mixed model) were detected in the vicinity of the mining industry (RR 1.240; 95%CI 1.124 - 1.368), refineries and coke ovens (RR 1.133; 95%CI 1.045 1.227), paper and wood production (RR 1.077; 95%CI 1.034 - 1.121), ship building (RR 1.064; 95%CI 1.014 - 1.117 ), fertiliser plants (1.060; 95%CI 1.008 - 1.115), food and beverage sector (RR 1.043; 95%CI 1.014 - 1.073), metal production and processing installations (RR 1.043; 95% CI 1.009 - 1.079) and ceramics (RR 1.036; 95%CI 1.002 - 1.072). The RR point estimates obtained using the BYM model, albeit similar to those of the mixed model, reached statistical significance in the vicinity of the mining industry (RR 1.258; 95%CI 1.082 - 1.463), paper and wood production (RR 1.071; 95%CI 1.007 – 1.140), food and beverage sector (RR 1.069; 95%CI 1.029 - 1.111), metal production and processing installations (RR 1.065; 95% CI 1.011 – 1.123) and ceramics (RR 1.050 ; 95%CI 1.004 - 1.099).

Among women, statistically significant excess mortality was detected by the two models in towns lying in the vicinity of ceramics (RR 1.085; 95%CI 1.024 - 1.150) and food and beverage production facilities (RR 1.070; 95%CI 1.018 - 1.124). Excess mortality was displayed by men living in towns near mining (RR 1.260; 95%CI 1.051 - 1.511), paper and wood production facilities (RR 1.088; 95%CI 1.010 - 1.173), surface treatment of metals and plastics (RR 1.049; 95%CI 1.000 - 1.100), and the food and beverage sector (RR 1.052; 95%CI 1.003 - 1.103) using the BYM model, and in these and other towns situated near refineries and coke ovens, glass and mineral fibre plants, and fertiliser industries using the mixed model.

The results of the sensitivity analysis with distances of 2, 3, 4, and 5 km for selected industrial sectors are shown in Table 3. The complete results for each sector are attached as Additional file 1.

**Table 3:** CRC mortality RR and 95% Credible Interval (CI) estimation in near vs. far analysis for selected industrial sectors for both sexes, men and women and different threshold distances

	**2 km**	**3 km**	**4 km**	**5 km**
	**RR 95% CI**	**RR 95% CI**	**RR 95% CI**	**RR 95% CI**
6 Mining industry	1.258	1.230	1.226	1.249
	1.082 - 1.463	1.065 - 1.420	1.078 - 1.395	1.103 - 1.414
Men	1.260	1.212	1.269	1.260
	1.051 - 1.511	1.026 - 1.432	1.088 - 1.479	1.086 - 1.462
Women	1.180	1.160	1.127	1.175
	0.976 - 1.428	0.964 - 1.395	0.956 - 1.328	1.003 - 1.377
20 Paper and wood production	1.071	1.078	1.065	1.066
	1.007 - 1.140	1.023 - 1.137	1.016 - 1.118	1.019 - 1.114
Men	1.088	1.062	1.066	1.075
	1.010 - 1.173	0.998 - 1.129	1.006 - 1.130	1.019 - 1.134
Women	1.031	1.037	1.062	1.049
	0.953 - 1.115	0.970 - 1.109	1.000 - 1.128	0.993 - 1.109
23 Food and beverage sector	1.069	1.067	1.067	1.072
	1.029 - 1.111	1.031 - 1.105	1.034 - 1.102	1.039 - 1.107
Men	1.052	1.036	1.067	1.075
	1.003 - 1.103	0.994 - 1.079	1.026 - 1.109	1.034 - 1.116
Women	1.070	1.053	1.059	1.062
	1.018 - 1.124	1.008 - 1.100	1.018 - 1.103	1.021 - 1.104
3 Metallurgical	1.065	1.057	1.064	1.073
	1.011 - 1.123	1.008 - 1.107	1.017 - 1.113	1.029 - 1.120
Men	1.059	1.023	1.068	1.080
	0.995 - 1.128	0.970 - 1.080	1.012 - 1.126	1.027 - 1.135
Women	1.033	1.018	1.037	1.042
	0.968 - 1.102	0.961 - 1.078	0.981 - 1.095	0.990 - 1.097
9 Ceramic	1.050	1.056	1.049	1.050
	1.004 - 1.099	1.014 - 1.100	1.009 - 1.091	1.012 - 1.089
Men	1.012	1.004	1.018	1.033
	0.956 - 1.071	0.956 - 1.055	0.969 - 1.069	0.987 - 1.082
Women	1.085	1.076	1.086	1.068
	1.024 - 1.150	1.021 - 1.134	1.032 - 1.142	1.020 - 1.120

## Discussion

The results of this exploratory study suggest that residing in the proximity of IPPC-registered industries with pollutant emissions to air could be a risk factor for CRC, inasmuch as both models detected higher mortality due to these tumours across the sexes for various industrial groups.

With respect to the results broken down by sex, attention should be drawn to the RRs (BYM model) registered for men in towns lying near mining industries, paper production, surface treatment of metals and plastics and the food and beverage sector, and for women in towns lying near ceramics and food and beverage production facilities. The excess mortality risk near refineries, mining industry, paper and wood production and the food and beverage sector was observed for men and women alike in the mixed model results. There are several types of industries that show differential outcomes by gender in the BYM model, namely: surface treatment of metals and plastics, and the mining industry in men; and ceramics in women. These sex-related differences could be indicative of underlying occupational exposures. Nevertheless, the occupational exposure component of these tumours has been little studied and described in the literature.

The rise in CRC incidence has paralleled socio-economic development in many countries. Hence, towns with industries nearby may have experienced greater socio-economic development than towns without industries, and it could be this that the results are reflecting. In this regard, our models include adjustment for socio-demographic variables such as income and proportion of unemployed, though this does not exclude residual confounding effects.

A critical question in study design is the choice of radius surrounding industrial installations. Our choice of 2 kilometres as the threshold distance in the “near vs. far” comparisons coincides with that used by other authors [27,28] and could be justified because, in these types of studies, if some increase in risk were to be found, it would most likely be in areas lying closest to the pollutant source. In CRC and other digestive system neoplasms, however, the population exposure pathway includes the food chain and there may possibly be no clear dilution of pollutants with increase of distance to the source. This can be observed by exploring the 2-, 3-, 4- and 5-kilometre thresholds in Table 3 (and Additional file 1), which shows no decrease in risk with the distance threshold for most of the industrial sectors that registered a statistically significant association. With the advance of environmental studies, there is increasingly more evidence of incorporation of different types of contaminants into the food chain, something that could be an explanation of the results associated with tumours of the digestive system [29-32].

We chose to analyse all tumours of the large intestine jointly, including unspecified tumours of the intestine, so as to avoid the difficulties posed by classification of tumours of the rectosigmoid junction (10% of cases). In Spain there is no incidence of a single cancer registry covering the whole territory. Mortality is thus the only universal source of information that can be used for an exploratory analysis such as ours. Furthermore, potential geographical disparities in CRC incidence are also expressed in mortality. Accordingly, this mortality study only considers a subset of CRC cases and so may conceivably have the effect of underestimating the impact of industrial sites on the occurrence of CRC.

Mortality rates depend on survival, and therefore on advances in medical technology. Mechanisms for disparities in cancer survival are multidimensional, vary according to the specific health care system involved, and may pertain to screening, diagnostic conditions, access to specialised care, treatment, or follow-up modalities, possibly inducing spatial heterogeneities in CRC mortality. In Spain, the 5-year CRC survival rate is 54% [33]. Since the Spanish National Health Service ensures equity in access to health care, there is no reason to believe that there would be health-care differences which might condition geographical disparities in mortality and also be related to proximity to pollutant sources [25].

The municipality is not a precise enough level of analysis to reflect exposure accurately. Since exposures of concern are related to air pollution, air dispersion models (or approximations) could be more relevant. Not all areas within a municipality are likely to be subjected to identical pollutant concentration levels. In addition, since industrial facilities are often located at the edge of municipalities, they are likely to affect residents of neighbouring facilities downwind. Unfortunately, meteorological covariates were not available for this analysis.

One of the chief strengths of this study is the use at a municipal level of a spatial hierarchical model which includes explanatory variables. The inclusion of spatial terms in the model, not only means that it is less susceptible to the possible presence of the ecological fallacy [34], but also ensures that the geographic heterogeneity of the distribution of mortality is taken into account. Although the results are not very different in the two models used, it should be mentioned that some estimators of RR may change sign depending on the model chosen, and in other cases the statistically significance of the association may disappear (e.g., refineries where the RR changes from 1.13 to 0.99). The use of the mixed model would be justified by its ease of adjustment and shorter computation time [35] but the method of estimation afforded by INLA amounts to a qualitative leap in the use of hierarchical models with explanatory variables. INLA is an alternative to Markov chain Monte Carlo methods, which furnish very similar results in far less computation time [36]. However, mixed models seem far more sensitive in detecting potential statistical relationships. Hierarchical models are perhaps too restrictive, and it has been reported that the conservative nature of their estimates rendered it advisable for the threshold of statistical significance to be reduced. The reasoning is as follows: since most environmental risks are small, these methods are seriously underpowered for the purpose of detecting them and, moreover, such methods are not suitable for localised excesses where the geographic source of the risk can be hypothesised, so that focused tests should be used instead [37]. In general, our results are noteworthy by virtue of the magnitude of the RR, since in ecological studies effect estimators for exposures such as environmental pollution tend to be very low.

Another strength of the study, apart from its statistical power, is the good quality of the information in terms both of diagnostic accuracy of cause of death in Spain [38] and quality of the inventory of pollutant industries. Reporting to the PRTR is compulsory by law. This means that to obtain an operating license, companies must report their activity and emissions to the Ministry for the Environment. The geographic co-ordinates used in this study were validated specifically [18].

In respect to the plausibility of results, whereas a Swedish study analysed occupational risk for colon cancer and concluded that occupation in general might play a small role in the aetiology of this tumour [39], two Canadian studies which analysed exposures to occupational agents and their relationship with colon [12] and rectal cancer [40], suggested the aetiological role of a series of industrial substances. Known aetiological factors in colorectal cancers include genetic predisposition, which would determine the presence of familial polyposis with tumours that very frequently become malignant. Hereditary factors are present in 10%-15% of cases, and other individual risk factors [41-43] or protective factors described are linked to dietary habits, lifestyle [44] and some medications (nonsteroidal anti-inflammatory drugs/NSAIDs, analgesics and statins) [45,46].

The mining-industry results warrant specific comment because this sector registered the highest RRs, exceeding 1.20. The sensitivity analysis for mining yielded very similar RR estimates at the different threshold distances explored (2, 3, 4 and 5 km). Of the 33 registered facilities, 12 were underground and 21 were opencast mines and quarries. Most of the metalloid and heavy metals (As, Cd, Cu, Cr, Ni, Pb and Zn) are released by these facilities into water. As already mentioned, the exposure pathway whereby these pollutants reach individuals might possibly be the trophic chain. This could explain why the distance to emission sources, at least as far as the 5-kilometre mark covered by our study, had no effect on the RR point estimates.

A recent study in the vicinity of a mine in Guangdong Province, China, with discharges to the environment for 30 years, has shown that the concentration of heavy metals in environmental samples was higher than in a reference area and that these values correlated with biological exposure markers in the population. The mortality rate ratios for all types of cancer were 2.13 and 2.83 in men and women respectively. Mortality rates were significantly increased for stomach, lung and oesophageal cancer in the high exposure area in comparison with the corresponding rates in the reference area, among men and women alike. The analysis showed that there were significantly positive correlations between exposure to cadmium and lead and the risk of all-cancer and stomach cancer mortality among women and both sexes. Unfortunately no results for colorectal cancer are shown [47].

Excess mortality in the proximity of metal production and processing facilities has already been described in an earlier study conducted with EPER data [13,14]. For a considerable number of years, it has been suspected that exposures deriving from work in the metal industry might possibly be related to tumours of the digestive system [48]. Indeed, there is evidence to show that exposure to metalworking fluids is associated with CRC [10] and, in the case of the galvanising sector, that dioxins are released during the passage of the metal through the molten zinc bath [49]. Even so, the results of our specific study into the galvanising sector, which we separated from the metal industry group precisely because of its dioxin emissions, indicated no association with colorectal cancer in men or women.

With regard to exposure to emissions from glass factories there is very little information. Cancer incidence studies targeting cohorts of glass workers in Sweden have reported a significant risk of CRC incidence exclusively among men [50]. Other studies have associated exposure to glass and mineral fibre with cancer of colon [12] and rectum [40], a finding that may be related with our results in respect of the glass sector.

Workers in the paper industry might have a higher risk of rectal cancer [51] but the information is very limited owing to the dearth of studies on occupation and CRC. A Japanese study reported elevated exposure to asbestos among patients with colon cancer who lived near a shipyard [52]. Moreover, occupational studies have already furnished evidence of excess risks of colon cancer among shipyard workers [53]. Some studies have shown that risk of CRC is higher among workers in the cement and fibre cement industry than among the general population [54,55], though other studies have failed to find this association [56]. For our part, we detected no increased risk in populations residing near the cement and lime industry.

The spatial pattern of CRC mortality in Spain reveals certain areas with higher risk, i.e., parts of Castile-León, the provinces of Barcelona and Girona, and, to a far more marked degree among women than among men, the provinces of Castellón, North Valencia, Alicante and Cadiz [25]. The Valencian Region is home to 36% of all ceramic factories. This region's mortality distribution pattern, coinciding with the local concentration of the ceramics industry, might thus be linked to the excess risk registered exclusively for women in the vicinity of these types of plants; and, though we are unaware of the breakdown by sex of the labour force in this particular sector, it has to be said that in some districts of Castellón the proportion of women workers has been shown to be considerably high [57].

## Conclusions

Our results should be evaluated with caution taking into account the exploratory nature of this study. To conclude that exposure to industrial emissions to air might be a risk factor for CRC, it would be necessary to confirm our findings with other studies. Nevertheless, some of the differences between men and women observed in the analyses of the different industrial groups suggest that there may be a component of occupational exposure, little-studied in the case of cancers of the digestive system.

## Competing interests

The authors declare that they have no competing interests

## Authors’ contributions

GLA, JGP, PFN, RR and EB were all involved in designing the study. GLA, PFN and JGP performed the statistical analysis. GLA wrote the first draft of the manuscript, to which all authors subsequently contributed. All authors made contribution to statistical analyses and interpretation of results, and revised the manuscript for important intellectual content. All authors read and approved the final manuscript.

## Pre-publication history

The pre-publication history for this paper can be accessed here:

http://www.biomedcentral.com/1471-2458/12/589/prepub

## Supplementary Material

Additional file 1 CRC mortality RR estimation and 95% credible interval (95%CI) in near vs far analysis for industrial sectors for both sexes, men and woman and diferent threshold distances. Click here for file
