# Supplementary material for: Colorectal cancer mortality and industrial pollution in Spain
Source: BMC Public Health. 2012 Aug 1;12:589. doi: 10.1186/1471-2458-12-589 (PMC3490748; doi:10.1186/1471-2458-12-589)
Supplement: Additional file 1 — CRC mortality RR estimation and 95% credible interval (95%CI) in near vs far analysis for industrial sectors for both sexes, men and woman and diferent threshold distances. [file 1471-2458-12-589-S1.pdf]

Suplementary material

CRC mortality RR estimation and 95% credible interval (95%CI) in near vs far analysis for industrial sectors for both sexes, men and woman and diferent threshold distances.

Industrial sector	2km	3km	4km	5km
	RR<br>(95% CI)	RR<br>(95% CI)	RR<br>(95% CI)	RR<br>(95% CI)
1 Combustion installations	1.028<br>0.962 1.099	1.036<br>0.982 1.093	1.059<br>1.011 1.109	1.066<br>1.020 1.114
Men	1.001<br>0.922 1.087	1.029<br>0.967 1.095	1.056<br>1.000 1.116	1.062<br>1.007 1.120
Women	1.076<br>0.986 1.174	1.039<br>0.972 1.110	1.049<br>0.991 1.110	1.053<br>0.997 1.112
2 Refineries and coke ovens	0.994<br>0.826 1.196	1.042<br>0.879 1.235	1.045<br>0.903 1.210	1.056<br>0.936 1.191
Men	0.999<br>0.806 1.238	1.080<br>0.896 1.302	1.064<br>0.894 1.265	1.043<br>0.905 1.202
Women	1.072<br>0.877 1.310	1.104<br>0.912 1.336	1.018<br>0.855 1.212	1.059<br>0.919 1.220
3 Metallurgical	1.065<br>1.011 1.123	1.057<br>1.008 1.107	1.064<br>1.017 1.113	1.073<br>1.029 1.120
Men	1.059<br>0.995 1.128	1.023<br>0.970 1.080	1.068<br>1.012 1.126	1.080<br>1.027 1.135
Women	1.033<br>0.968 1.102	1.018<br>0.961 1.078	1.037<br>0.981 1.095	1.042<br>0.990 1.097
4 Galvanization	1.037<br>0.958 1.123	1.055<br>0.984 1.130	1.044<br>0.980 1.112	1.040<br>0.982 1.102
Men	1.048<br>0.952 1.153	1.032<br>0.954 1.116	1.055<br>0.979 1.137	1.040<br>0.971 1.114
Women	0.993<br>0.896 1.099	1.011<br>0.928 1.101	1.015<br>0.939 1.097	1.030<br>0.959 1.106
5 Surface treatment of metals and plastic	1.037<br>0.997 1.079	1.054<br>1.017 1.092	1.063<br>1.026 1.101	1.062<br>1.026 1.100
Men	1.049<br>1.000 1.100	1.022<br>0.980 1.065	1.067<br>1.022 1.113	1.063<br>1.019 1.109
Women	0.984<br>0.938 1.034	1.011<br>0.968 1.056	1.039<br>0.995 1.085	1.042<br>0.999 1.088
6 Mining industry	1.258<br>1.082 1.463	1.230<br>1.065 1.420	1.226<br>1.078 1.395	1.249<br>1.103 1.414
Men	1.260<br>1.051 1.511	1.212<br>1.026 1.432	1.269<br>1.088 1.479	1.260<br>1.086 1.462
Women	1.180<br>0.976 1.428	1.160<br>0.964 1.395	1.127<br>0.956 1.328	1.175<br>1.003 1.377
7 Cement and lime	0.977<br>0.884 1.080	1.025<br>0.944 1.114	1.051<br>0.985 1.122	1.055<br>0.995 1.118
Men	0.928<br>0.818 1.053	0.949<br>0.857 1.051	0.991<br>0.915 1.075	1.009<br>0.940 1.083
Women	1.049<br>0.921 1.194	1.099<br>0.987 1.223	1.137<br>1.048 1.233	1.115<br>1.037 1.199
8 Glass and mineral fibres	1.077<br>0.977 1.187	1.047<br>0.976 1.124	1.063<br>0.995 1.136	1.075<br>1.011 1.142

Men	1.105<br>0.982 1.243	1.036<br>0.956 1.122	1.036<br>0.957 1.122	1.050<br>0.977 1.128
Women	1.031<br>0.907 1.172	1.017<br>0.933 1.108	1.090<br>1.006 1.182	1.092<br>1.016 1.174
9 Ceramic	1.050<br>1.004 1.099	1.056<br>1.014 1.100	1.049<br>1.009 1.091	1.050<br>1.012 1.089
Men	1.012<br>0.956 1.071	1.004<br>0.956 1.055	1.018<br>0.969 1.069	1.033<br>0.987 1.082
Women	1.085<br>1.024 1.150	1.076<br>1.021 1.134	1.086<br>1.032 1.142	1.068<br>1.020 1.120
10 Organic chemical industry	1.011<br>0.958 1.067	1.033<br>0.987 1.082	1.038<br>0.994 1.083	1.053<br>1.010 1.098
Men	1.015<br>0.951 1.083	1.019<br>0.966 1.074	1.054<br>1.001 1.110	1.066<br>1.013 1.121
Women	0.979<br>0.915 1.049	0.989<br>0.935 1.047	1.009<br>0.957 1.064	1.022<br>0.971 1.076
11 Inorganic chemical industry	0.962<br>0.882 1.049	1.013<br>0.954 1.077	1.027<br>0.971 1.085	1.041<br>0.989 1.096
Men	0.911<br>0.820 1.013	0.979<br>0.913 1.050	1.029<br>0.963 1.100	1.046<br>0.983 1.113
Women	1.011<br>0.907 1.127	0.994<br>0.922 1.073	1.013<br>0.945 1.086	1.021<br>0.958 1.089
12 Fertilisers	1.073<br>0.978 1.178	1.053<br>0.965 1.149	1.052<br>0.969 1.142	1.049<br>0.971 1.133
Men	1.098<br>0.983 1.227	1.075<br>0.975 1.186	1.101<br>0.999 1.214	1.075<br>0.980 1.179
Women	1.022<br>0.916 1.140	0.993<br>0.896 1.101	0.988<br>0.894 1.091	1.014<br>0.923 1.113
13 Biocides	1.125<br>0.977 1.295	1.048<br>0.934 1.175	1.088<br>0.976 1.212	1.064<br>0.959 1.181
Men	1.117<br>0.937 1.331	1.001<br>0.873 1.148	1.084<br>0.949 1.240	1.066<br>0.937 1.212
Women	1.077<br>0.891 1.302	1.016<br>0.872 1.182	1.052<br>0.910 1.216	1.029<br>0.895 1.184
14 Pharmaceutical products	1.052<br>0.978 1.131	1.041<br>0.983 1.103	1.048<br>0.990 1.110	1.077<br>1.019 1.138
Men	1.037<br>0.950 1.132	1.014<br>0.950 1.083	1.048<br>0.979 1.122	1.079<br>1.010 1.152
Women	1.039<br>0.952 1.134	1.010<br>0.943 1.081	1.036<br>0.968 1.109	1.052<br>0.985 1.123
15 Explosives and pyrotechnics	0.983<br>0.895 1.080	1.027<br>0.951 1.109	1.028<br>0.957 1.104	1.017<br>0.949 1.089
Men	0.990<br>0.881 1.113	1.011<br>0.923 1.108	1.009<br>0.924 1.102	0.995<br>0.914 1.083
Women	0.984<br>0.870 1.112	1.035<br>0.938 1.141	1.049<br>0.958 1.149	1.042<br>0.955 1.138
16 Hazardous waste	1.014<br>0.935 1.100	1.062<br>1.002 1.125	1.060<br>1.007 1.116	1.073<br>1.023 1.126
Men	1.010<br>0.914 1.117	1.042<br>0.975 1.113	1.086<br>1.021 1.155	1.102<br>1.041 1.166

Women	1.017<br>0.917 1.128	1.032<br>0.962 1.106	1.018<br>0.955 1.085	1.027<br>0.969 1.090
17 Non-hazardous waste	1.000<br>0.898 1.115	1.073<br>1.003 1.148	1.107<br>1.044 1.175	1.121<br>1.066 1.179
Men	1.011<br>0.888 1.152	1.072<br>0.994 1.157	1.156<br>1.078 1.239	1.157<br>1.090 1.228
Women	0.963<br>0.839 1.105	1.005<br>0.926 1.092	1.030<br>0.956 1.110	1.056<br>0.991 1.124
18 Disposal or recycling of animal waste	1.019<br>0.903 1.150	1.038<br>0.948 1.137	1.013<br>0.942 1.089	1.029<br>0.963 1.099
Men	0.971<br>0.833 1.132	1.005<br>0.904 1.117	0.998<br>0.913 1.091	1.027<br>0.947 1.113
Women	1.069<br>0.910 1.255	1.032<br>0.924 1.154	1.034<br>0.946 1.132	1.035<br>0.953 1.124
19 Urban waste-water treatment plants	1.055<br>0.986 1.129	1.038<br>0.987 1.093	1.047<br>1.000 1.095	1.051<br>1.007 1.096
Men	1.006<br>0.926 1.093	0.994<br>0.937 1.055	1.030<br>0.975 1.089	1.036<br>0.984 1.091
Women	1.080<br>0.995 1.172	1.026<br>0.965 1.091	1.054<br>0.998 1.113	1.054<br>1.001 1.110
20 Paper and wood production	1.071<br>1.007 1.140	1.078<br>1.023 1.137	1.065<br>1.016 1.118	1.066<br>1.019 1.114
Men	1.088<br>1.010 1.173	1.062<br>0.998 1.129	1.066<br>1.006 1.130	1.075<br>1.019 1.134
Women	1.031<br>0.953 1.115	1.037<br>0.970 1.109	1.062<br>1.000 1.128	1.049<br>0.993 1.109
21 Pre-treatment or dyeing of textiles	1.015<br>0.914 1.128	1.007<br>0.908 1.117	1.029<br>0.933 1.136	1.010<br>0.921 1.106
Men	1.019<br>0.893 1.164	0.987<br>0.869 1.121	1.019<br>0.900 1.154	1.006<br>0.897 1.128
Women	0.987<br>0.857 1.136	0.988<br>0.861 1.135	1.028<br>0.901 1.172	1.000<br>0.885 1.130
23 Food and beverage sector	1.069<br>1.029 1.111	1.067<br>1.031 1.105	1.067<br>1.034 1.102	1.072<br>1.039 1.107
Men	1.052<br>1.003 1.103	1.036<br>0.994 1.079	1.067<br>1.026 1.109	1.075<br>1.034 1.116
Women	1.070<br>1.018 1.124	1.053<br>1.008 1.100	1.059<br>1.018 1.103	1.062<br>1.021 1.104
26 Surface treatment using organic solvents	1.055<br>0.989 1.126	1.066<br>1.013 1.121	1.072<br>1.024 1.123	1.072<br>1.027 1.120
Men	1.077<br>0.995 1.164	1.042<br>0.983 1.104	1.081<br>1.023 1.142	1.084<br>1.028 1.142
Women	1.000<br>0.923 1.085	1.008<br>0.947 1.072	1.042<br>0.985 1.102	1.039<br>0.985 1.096
28 Ship building	1.093<br>0.948 1.261	1.103<br>0.962 1.264	1.106<br>0.965 1.268	1.107<br>0.965 1.269
Men	1.102<br>0.936 1.299	1.114<br>0.964 1.288	1.126<br>0.961 1.319	1.126<br>0.962 1.319
Women	1.072<br>0.932 1.233	1.075<br>0.938 1.232	1.076<br>0.934 1.240	1.077<br>0.934 1.240
